# A novel apoE-mimetic increases brain apoE levels, reduces Aβ pathology and improves memory when treated before onset of pathology in male mice that express *APOE3*

**DOI:** 10.1186/s13195-023-01353-z

**Published:** 2023-12-15

**Authors:** Ana C. Valencia-Olvera, Deebika Balu, Shreya Bellur, Thomas McNally, Yaseen Saleh, Don Pham, Shivesh Ghura, Jason York, Jan O. Johansson, Mary Jo LaDu, Leon Tai

**Affiliations:** 1 Department of Anatomy and Cell Biology, University of Illinois at Chicago, Chicago, IL USA; 2 Department of Surgery, University of Washington, Seattle, WA USA; 3 University of Miami/Jackson Healthcare System, Miami, FL USA; 4 Department of Dentistry, University of Illinois at Chicago, Chicago, IL USA; 5 Department of Pharmacology, University of Pennsylvania, Philadelphia, PA USA; 6 Artery Therapeutic’s Inc, San Ramon, CA USA

**Keywords:** Alzheimer’s disease, apoE, CS-6253, EFAD mice

## Abstract

**Background:**

Alzheimer’s disease (AD) is characterized by cognitive dysfunction and amyloid plaques composed of the amyloid-beta peptide (Aβ). *APOE* is the greatest genetic risk for AD with *APOE4* increasing risk up to ~ 15-fold compared to *APOE3.* Evidence suggests that levels and lipidation of the apoE protein could regulate AD progression. In glia, apoE is lipidated via cholesterol efflux from intracellular pools, primarily by the ATP-binding cassette transporter A1 (ABCA1). Therefore, increasing ABCA1 activity is suggested to be a therapeutic approach for AD. CS-6253 (CS) is a novel apoE mimetic peptide that was developed to bind and stabilize ABCA1 and maintain its localization into the plasma membrane therefore promoting cholesterol efflux. The goal of this study was to determine whether CS could modulate apoE levels and lipidation, Aβ pathology, and behavior in a model that expresses human *APOE* and overproduce Aβ.

**Methods:**

In vitro, *APOE3*-glia or *APOE4*-glia were treated with CS. In vivo, male and female, E3FAD (5xFAD^+/−^/*APOE3*^+/+^) and E4FAD (5xFAD^+/−^/*APOE4*^+/+^) mice were treated with CS via intraperitoneal injection at early (from 4 to 8 months of age) and late ages (from 8 to 10 months of age). ApoE levels, ABCA1 levels and, apoE lipidation were measured by western blot and ELISA. Aβ and amyloid levels were assessed by histochemistry and ELISA. Learning and memory were tested by Morris Water Maze and synaptic proteins were measured by Western blot.

**Results:**

CS treatment increased apoE levels and cholesterol efflux in primary glial cultures. In young male E3FAD mice, CS treatment increased soluble apoE and lipid-associated apoE, reduced soluble oAβ and insoluble Aβ levels as well as Aβ and amyloid deposition, and improved memory and synaptic protein levels. CS treatment did not induce any therapeutic benefits in young female E3FAD and E4FAD mice or in any groups when treatment was started at later ages.

**Conclusions:**

CS treatment reduced Aβ pathology and improved memory only in young male E3FAD, the cohort with the least AD pathology. Therefore, the degree of Aβ pathology or Aβ overproduction may impact the ability of targeting ABCA1 to be an effective AD therapeutic. This suggests that ABCA1-stabilizing treatment by CS-6253 works best in conditions of modest Aβ levels.

**Supplementary Information:**

The online version contains supplementary material available at 10.1186/s13195-023-01353-z.

## Background

Alzheimer’s disease (AD) is a fatal neurodegenerative disease and the primary cause of age-related dementia [1]. AD is characterized by cognitive dysfunction and amyloid plaques composed of the amyloid-beta peptide (Aβ) in addition to other pathologies [2]. Current AD treatments include acetylcholinesterase inhibitors, and glutamate regulators that only ameliorate the symptoms and the recently approved Aβ-immunotherapies aducanumab and lecanemab [3]. One alternative approach is to focus on pathways modulated by known AD risk factors. *APOE* is the greatest genetic risk factor for AD, with *APOE4* increasing risk up to 15-fold compared to *APOE3* [4]. In this study, we evaluated whether pharmacologically targeting apoE protein levels/structure could be a beneficial approach for mitigating AD pathology in vivo.

ApoE is the major lipoprotein-competent apolipoprotein in the central nervous system (CNS) and is primarily produced by astrocytes [5–7]. ApoE production, modification, trafficking, and transport pathways are complex and not fully understood [8, 9]. However, in general, apoE is thought to become post-translationally lipidated to then induce multiple functional effects, e.g., lipid trafficking, metabolism, inflammation, signaling, and regulation of Aβ levels [10–12]. Importantly, apoE4 levels are thought to be lower, less lipidated, and less stable compared to apoE3 in humans, in vivo and in vitro [13–17]. However, whether modulating apoE4 levels and/or lipidation is beneficial for regulating Aβ levels is conflicted. Indeed, studies that have found that both decreasing and increasing apoE4 levels/lipidation can lower Aβ deposition in FAD-tg mice [8, 9, 18–25]. Thus, there are different views on how to therapeutically target apoE4 to lower Aβ levels. One view is that apoE4 is detrimental, and therefore the optimal approaches are to lower apoE4 levels, correct the structure of apoE4, or replace apoE4 with other genotypes using viral vectors [19–22, 26–28]. An alternative view is that increasing apoE transcription [9, 18, 29] or apoE lipidation, which could also result in higher levels through increasing lipoprotein stability [30], could be beneficial.

In glia, apoE is lipidated via cholesterol efflux from intracellular pools, primarily by the ATP-binding cassette transporter A1 (ABCA1) [31, 32]. Interestingly, overexpressing ABCA1 reduces Aβ pathology, while deletion of ABCA1 increases Aβ pathology in vivo [33, 34]. Developed by Artery Therapeutics, CS-6253 (EVC*SKLEEWLAALC*ELAEE-LLARLKS, where C* are citrulines) is a small α-helical peptide derived from the C-terminal lipid-binding domain of apoE, a region that has been shown to stimulate cholesterol efflux via ABCA1 [35–37]. As it was originally developed for atherosclerosis treatment, CS-6253 (CS) has shown to offer lipidemic and metabolic benefits in atherogenic models like *APOE*-Knockout (KO) and DIO ob/ob mice [36, 38]. Importantly, previous studies in mice without Aβ pathology, α-synuclein-KO/*APOE4-targeted replacement* mice, have shown that CS can increase brain apoE lipidation and cognitive performance [24]. Although this data suggests that targeting ABCA1 with CS is an attractive approach, there are unresolved questions related to this strategy in an AD-relevant context. One is whether targeting ABCA1 is sufficient to increase apoE4 levels and lipidation. For example, the detrimental effects of *APOE4* may limit the extent that ABCA1 can transfer cholesterol and/or apoE4 may not be capable of accepting more cholesterol from ABCA1 without additional lipoprotein modifications [39]. Thus, it has been proposed that targeting ABCA1 may be more beneficial for apoE3. Second, and related, is whether the presence of high levels of human Aβ modulates the ability of ABCA1 to induce beneficial effects [40]. To date, no studies have evaluated the activity of CS on models that express human apoE and high human Aβ levels.

The goal of this study was to determine whether CS could modulate apoE levels and lipidation, Aβ pathology, and behavior in vivo. To address this goal, we treated mice that express human apoE3 or apoE4 and overproduce Aβ with CS and evaluated the impact on AD-relevant pathology and behavior.

## Materials and methods

### CS preparation

The CS-6253 (CS) peptide was synthesized and provided by Artery Therapeutics, Inc. at 98% purity. The lyophilized peptide was dissolved in Dulbecco’s phosphate buffer solution (DPBS) (pH = 7.4).

### In vitro

#### Glial cultures

**Fig. 1:**
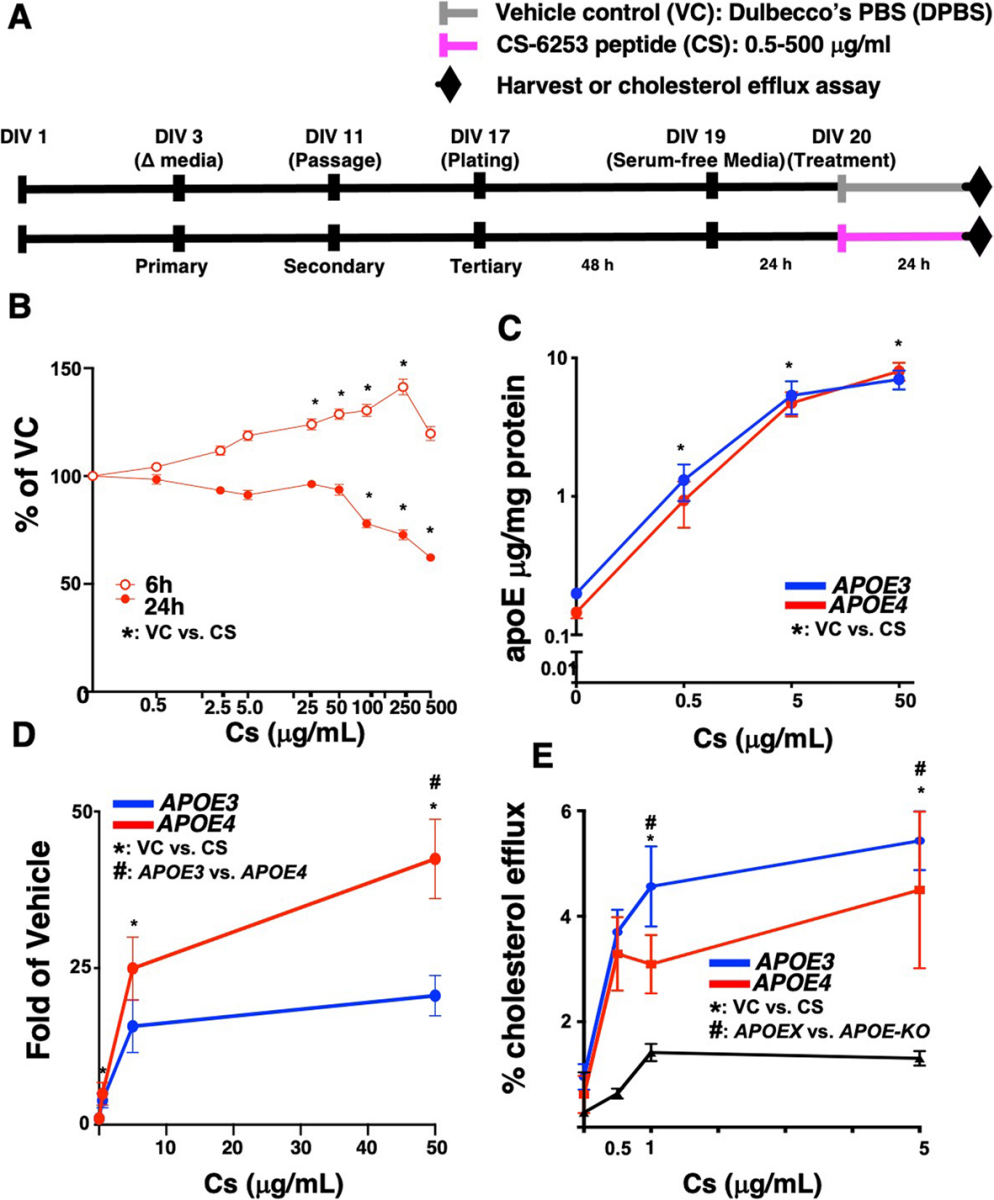
CS treatment increases apoE secretion and cholesterol efflux from *APOE3* and *APOE4* cultured glia.** A** In vitro study design. Mixed glial were treated with CS (0.5–500 μg/ml) or vehicle control (VC) for 6, 12, or 24h. **B** Cell viability of *APOE4*-glia in response to 6-h or 24-h treatment with increasing concentrations of CS (0.0–500 μg/ml) measured by MTT assay. There was an interaction between concentration and treatment time [*F*_(8,54)_: 20.86, *p* < 0.0001] because compared to vehicle, CS lowered MTT signal beyond 100 μg/ml with 24-h treatment (100: *p* = 0.0101; 250: *p* = 0.0003; 500: *p* < 0.0001), however, increased signal at concentrations above 25 μg/ml with 6-h treatment (25: *p* = 0.0029; 50: *p* = 0.0001; 100: *p* < 0.0001; 500: *p* < 0.0463). **C** Quantification of apoE secreted by *APOE3-* or *APOE4-*glia in response to 24-h CS treatment (0.0–50.0 μg/ml) when measured by ELISA (media). CS treatment increased apoE levels [treatment, *F*_(3,24)_: 29.55, *p* < 0.0001]. **D** Quantification of fold increase in secreted apoE levels (ELISA, media) by glia treated with CS (50 μg/ml) for 6, 12, and 24 h. Relative apoE levels in the media are greater with *APOE4*-glia at 24 h (*p* = 0.0002). **E** The effect of CS on cholesterol efflux capacity. *APOE-KO*, *APOE3-*, or *APOE4*-glial were treated with CS for 24 h (0.0–5.0 μg/ml), and the media was added to *APOE-KO*-glia loaded with BODIPY-cholesterol. CS treatment promoted cholesterol efflux [Treatment effect: *F*_(3,29)_: 9.27, *p* = 0.0002], with *APOE3-* and* APOE4- *but not* APOE-KO-*glia [Genotype effect: *F*_(3,29)_: 13.00, *p* < 0.0001]. Data are expressed as means ± SEM (*n* = 4) and were analyzed by 2-way (B, C, D) or 3-way ANOVA (1D), followed by Tukey postdoc: *p* < 0.05

Primary mixed glial cultures (~ 95% astrocytes, 5% microglia) were isolated from the cerebral cortex of 2-day old E3FAD and E4FAD non-carrier (5xFAD^−/−^/*APOE3*^+/+^ and 5xFAD^−/−^/*APOE4*^+/+^) pups as described in [41, 42]. Glia reached confluency after 10–12 days in vitro (DIV) and were trypsinized and plated into two 175-cm^2^ tissue culture flasks. After 20 DIV, secondary mixed glial cultures were seeded on a 96-well plate. Twenty-four hours (h) after replacement of media without serum, cells were treated with varying concentrations of CS for 24 h (Fig. 1A).

#### Cholesterol efflux assay

Cholesterol efflux studies were conducted as previously described [43]. Briefly, *APOE-*Knockout (KO) mixed glial cultures were incubated overnight at 37 °C with labeling reagent (BODIPY-cholesterol). Following a wash (RPMI), *APOE*-KO-glia were incubated with conditioned media from CS-treated (0–10 µg/mL) *APOE3*-glia or *APOE4*-glia (37 °C for 4–6 h), and the fluorescence in the media was measured (Ex/Em = 482/515 nm). One hundred microliters of RIPA Lysis buffer was added to the resulting cell monolayer and shaken for 30 min at room temperature (RT), after which fluorescence in the cell lysate was measured (Ex/Em = 482/515 nm). Cholesterol efflux values were reported as a percentage of fluorescence intensity of the media divided by fluorescence intensity of cell lysate and media. Note. Fluorescence was measured in black-walled 96-well plates.

#### MTT for cell viability

After treatment with CS for 6 h and 24 h, the media was removed, and the cells incubated with 5% 3-(4,5-dimethylthiazol-2-yl)-2,5-diphenyltetrazolium bromide (MTT) and processed as per the manufacturer’s instructions. The absorbance values at 595 nm are expressed as % of vehicle control (VC).

#### ApoE ELISA

ApoE levels were measured in the media of glial cell cultures with an in-house ELISA. The ELISA uses an α-apoE capture antibody (1:2000, Millipore) and a biotinylated α-apoE detection antibody (1:5000, Meridian) as described in [20]. A recombinant human apoE3 standard was prepared and diluted to produce the standard curves (695H). Samples were diluted to read within the range of the standard curve (4-parameter logistic regression).

### In vivo

#### EFAD mice development, care, and use

Experiments followed the University of Illinois at Chicago Institutional Animal Care and Use Committee protocols. To establish colonies of EFAD mouse lines, 5xFAD mice (APP K670N/M671L + I716V + V717I and PS1 M146L + L286V, clone Tg6799) on a C57B//B6xSJL background were bred to homozygous *APOE3*-, and *APOE4*-TR mice on a C57/B6 background by Taconic Laboratories. EFAD have been maintained at the UIC as in inbred strain for ~ 10 years. EFAD mice are hemizygous for 5xFAD genes (5xFAD^+/−^) and homozygous for *APOE*. Therefore, for breeding we use either male 5xFAD^+/−^*APOE*X^+/+^ and female 5xFAD^−/−^*APOE*X^+/+^ mice or male 5xFAD^−/−^*APOE*X^+/+^ and female 5xFAD^+/−^*APOEX*^+/+^ mice. All breeding and colony maintenance was conducted at UIC Biologic Resources Laboratory as previously described [14]. Mice were weaned at P21, ear tagged, and genotyped for 5xFAD. Cages contained four to five mice at the start of housing, which continued unless there was a premature loss of mice due to natural causes, fighting prior to the start of experiments or during treatment. For cohort assignment, around half of the mice of each cage were randomly assigned to vehicle control (VC) or CS-6253 treatment (CS). For treatment, experiments, and quantification, investigators were blinded to CS treatment.

#### Drug treatment

**Fig. 2:**
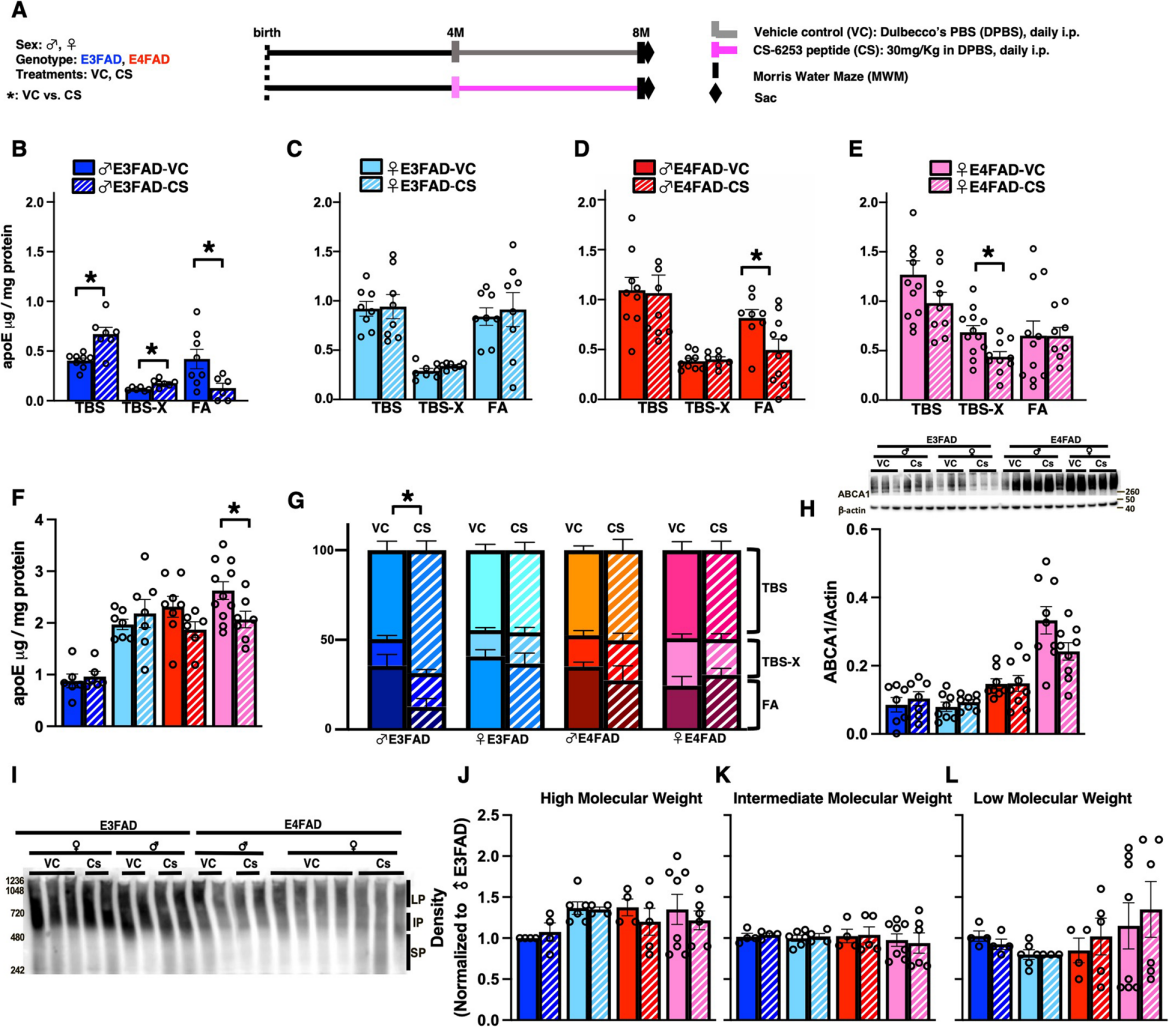
CS treatment modifies apoE levels in male E3FAD mice. **A** In vivo study design. EFAD mice were treated with either vehicle control (VC) or CS by daily intraperitoneal (i.p.) injection from 4 to 8 months of age. **B**–**G** Cortical proteins were serially extracted in TBS (soluble), 1% TritonX100 (detergent soluble), and formic acid (insoluble) and apoE levels were measured by ELISA. CS treatment modulated apoE levels only in male E3FAD mice. **B** CS treatment increased soluble (*p* = 0.0017) and detergent-soluble apoE (*p* = 0.0028) and decreased insoluble apoE (*p* = 0.0323) in male E3FAD mice. **C** ApoE levels were not affected by CS treatment in female E3FAD mice. **D** CS treatment reduced insoluble apoE levels in male E4FAD mice (*p* = 0.0234). **E** CS treatment reduced detergent-soluble apoE levels in female E4FAD mice (*p* = 0.0108). **F** There was a reduction with CS treatment in total apoE levels in female E4FAD (*p* = 0.0397). **G** ApoE levels graphed as parts of a whole. ApoE distribution across extracts was modified by CS treatment in male E3FAD (*p* < 0.0001, analyzed by chi-square (and Fisher’s exact test) *: *p* < 0.0001.** H** ABCA1 levels quantified by western blot in the detergent fraction. There were no effects of CS treatment. **I** Representative image of native gels for apoE measured in the soluble fraction. **J**–**L** Quantification of larger (high molecular weight, LP), intermediate (intermediate molecular weight, IP), and small (low molecular weight, SM) apoE particles. There was no effect on levels of LP, IP, or SP with CS treatment. Data are expressed as mean ± SEM (*n* = 8–12), analyzed by *t*-test, *: *p* < 0.05 CS vs. VC. All experiments were done in the cortex. All data is from the same cohort of mice; the n’s differ because of study design, technical issues, and outlier tests

Male and female, E3FAD and E4FAD mice were treated with CS (30 mg per kg) or vehicle control (DPBS) via daily intraperitoneal injection in either an early paradigm (from 4 to 8 months of age, Fig. 2A) or late paradigm (from 8 to 10 months of age, Fig. 6A). Body weights were collected every day before treatment to assure a precise dose. *N* = 12 per cohort.

#### Behavioral analysis (MWM)

All behavioral data were recorded and analyzed with ANY-maze video tracking software (Stoelting Co, Wood Dale, IL USA). In the week prior to sacrifice, mouse behavior was evaluated using an adapted Morris water maze (MWM) protocol (previously described [44]). Briefly, acquisition trials (learning) consisted of 4 × 1-min trials/day for 5 consecutive days with latency to the platform recorded for each trial. A single probe trial was run on day 6 with the platform removed, and the readouts included latency to platform, latency to target quadrant, time spent in target quadrant, and the number of platform crosses. After the probe trial, mice were euthanized as described below.

#### Tissue harvest

Mice were anesthetized via i.p. injection with ketamine (100 mg/kg) and xylazine (10 mg/kg) and transcardially perfused with ice-cold PBS with protease inhibitors. Whole livers were extracted, weighted, and flash-frozen in liquid nitrogen. Right hemispheres were dissected into cortex, flash-frozen in liquid nitrogen, and stored at – 80 °C until processing. Left hemispheres were fixed in 4% paraformaldehyde for 48 h and stored in 0.05% sodium azide in PBS at 4°C as previously described [14].

#### Sequential protein extraction

Cortices were processed using a three-step-sequential protein fractionation method, resulting in soluble (Tris-buffered saline: TBS), non-ionic detergent (TBS + 1%Triton X-100: TBSX), and insoluble (neutralized formic acid: FA) proteins as previously described [45]. Soluble (TBS) and insoluble (FA) total protein was quantified by Bradford and detergent soluble total proteins (TBSX) were quantified by BCA Protein Assay as previously described [45].

#### ELISA

Soluble oAβ and Aβ24 levels were measured in TBS brain homogenates (biosensis and Life Technologies, respectively) and insoluble Aβ42 (Life Technologies) in the FA fraction. ApoE levels were measured in TBS, TBSX, and FA cortex homogenates by ELISA as in vitro experiments (see above) [20]. All samples were diluted to fit within the sensitivity of each ELISA.

#### Western blot analyses

Western blots were conducted as previously described [44]. Briefly, 15 μg of total protein of brain homogenates were incubated with loading buffer, heated in a sand bath for 5 min at 90 °C, and loaded into wells of 4–12% Bis–Tris NuPAGE precast gels (Invitrogen). Following electrophoresis, proteins were transferred onto 0.2μm PVDF membranes (Invitrogen), incubated in blocking buffer (5% nonfat dry milk in TBS + 0.065%Tween-20) for 1h, and with primary antibodies in blocking buffer at 4 °C overnight. Membranes were then washed in TBS + Tween-20, incubated with HRP-conjugated secondary antibodies for 45 min, washed again, developed with Pierce chemiluminescence reagents, and visualized with an Odyssey FC Imaging System. For antibody use, see Supplementary Table 1.

#### Native gels

For native gels, 15 μg of protein from TBS fractions was separated on 4–20% Tris–glycine gels following the manufacturer’s instructions and transferred to 0.2μm PVDF at 30 V for 16h. After transfer, blots were treated with Ponceau S Staining Solution (0.1% (w/v) Ponceau S in 5% (v/v) acetic acid) to visualize the molecular mass markers (pierce). Membranes were incubated in 5% nonfat dry milk in TBS for 1h and incubated overnight with primary goat anti-apoE antibody in 1% nonfat dry milk overnight at 4 °C, followed by HRP-conjugated secondary antibodies for 45 min in 1% nonfat dry milk, developed with Pierce chemiluminescence reagents, and visualized with an Odyssey FC Imaging System as previously described (see Supplementary Table 1) [16].

#### Immunohistochemical analysis

Serial sagittal brain sections (35-μm thick, 280 μm apart) from EFAD mice were all stained for fibrillar amyloid deposition via Thio-S, and immunostained for Aβ deposition (MOAB2, *in house*) as previously described [14], with anti-mouse or anti-rabbit Alexa-fluor secondary antibodies (Supplementary Table 1). The stained sections were imaged at 10 × magnification with a Zeiss Fluorescence microscope and analyzed for the area covered by Thio-S and MOAB-2 in the cortex using ImageJ. Signals from cortical regions were quantified by investigators blinded to treatment, *APOE* genotype, and sex within the paradigm.

### Statistical analysis

GraphPad Prism 9 (for Mac, GraphPad Software, La Jolla, CA) was used for outlier tests (ROUT 10%), two-way analysis of variance (ANOVA) followed by Tukey’s post hoc tests, *χ*^2^, or Student’s* t*-test. Data was plotted as scatter bar graphs, with the mean and standard error of the mean (SEM).

## Results

### CS treatment increases apoE secretion and cholesterol efflux in mixed glial cultures

The premise of this study is that CS-6253 (CS) treatment can increase apoE levels and/or the capacity of apoE to accept cholesterol. Therefore, we initially tested whether CS modulates apoE secretion and cholesterol efflux in vitro, using mixed glial cultures isolated from E3FAD and E4FAD non-carrier (*APOE*x-glia, Fig. 1A).

At non-toxic concentrations (Fig. 1B and raw values in Supplementary Fig. 1A), CS treatment (0.5, 5 and 50 μg/mL for 24 h) increased apoE levels in the media for both *APOE* genotypes (Fig. 1C). CS itself is not detected by the apoE ELISA (Supplementary Fig. 1B). Interestingly, the relative increase in apoE levels was greater in *APOE4*-glia than *APOE3-*glia at 50 μg/ml CS treatment (Fig. 1D). To measure cholesterol efflux, *APOE3*-glia and *APOE4*-glia were treated with 3 concentrations of CS (0.5, 1.0, and 5.0 μg/mL) for 24 h and the conditioned media added to BODIPY-Cholesterol loaded *APOEKO*-glia (Fig. 1E). CS increased cholesterol efflux at 5 μg/mL for both *APOE* genotypes (Fig. 1E). Interestingly, in *APOE-KO* glia treated with CS there was a marginal increase of cholesterol efflux, suggesting that CS itself could act as a cholesterol acceptor when there are no apolipoproteins present as previously shown [36]. However, the cholesterol efflux by CS on *APOE-KO* glia was much lower than *APOE3* and *APOE4* treated glia. Thus, CS increased apoE3 and apoE4 levels and cholesterol efflux in vitro.

### CS treatment increases soluble and detergent soluble apoE levels in male E3FAD mice

Our overall goal was to evaluate the effect of CS-6253 (CS) treatment on apoE levels, AD-relevant pathology, and behavior in mice that express human *APOE*. Therefore, we utilized EFAD mice that express human *APOE4* (E4FAD) or *APOE3* (E3FAD) and overproduce Aβ42. We treated male and female, E3FAD and E4FAD mice with either vehicle (vehicle control) or CS from 4 to 8 months of age (30 mg/kg, intraperitoneal injection every day) (Fig. 2A). We selected 4 months to start treatment at early stages of *APOE*-modulated pathology. Indeed, we corroborated and expanded on previous findings that at 4 months of age, compared to E3FAD mice, E4FAD mice have lower levels of detergent-soluble apoE (an indication of lipid-associated apoE), higher Aβ pathology, and impaired learning and memory in the Morris water maze (MWM) test [14] (Supplementary Fig. 2). We used 30 mg/kg CS as previous in vivo studies have reported beneficial effects on different readouts around that dose, i.e., at 20 mg/kg every other day [24]. Our focus was evaluating whether CS treatment resulted in a beneficial effect, and therefore statistical analysis was primarily conducted using within-group comparisons, i.e., comparing vehicle control (VC) with CS within a genotype and sex.

First, we evaluated markers of indirect target engagement, including apoE and ABCA1 levels as well as apoE lipidation in the cortex of EFAD mice. To measure apoE levels (ELISA) we performed a 3-step sequential protein extraction (TBS, 1% Triton X-100, and then formic acid) from the cortex [45] (Fig. 2B–G). The sequential protein extraction was developed to quantify the size of intact lipoproteins (native gels of TBS extract), the amount of lipid-associated apoE through extraction with 1%Triton X-100, and the levels of insoluble apoE (formic acid, plaque associated) [45]. We found CS treatment increased soluble and detergent-soluble apoE levels by 60% and decreased insoluble apoE levels by 70% in male E3FAD mice (Fig. 2B). Interestingly, CS treatment lowered insoluble apoE levels in male E4FAD mice (Fig. 2D) and detergent-soluble apoE levels in female E4FAD mice (Fig. 2E). Confirming these data, CS treatment markedly changed the distribution of apoE among fractions in male E3FAD (Fig. 2G). We next explored whether CS affects the lipidation of apoE-lipoproteins by measuring ABCA1 levels by western blot and apoE lipoprotein size by native gels (Fig. 2H–L). Unexpectedly, CS treatment did not impact levels of ABCA1 or the size of apoE lipoproteins in any group.

Collectively our data demonstrated that CS treatment increases soluble and detergent soluble apoE levels and decreased insoluble apoE in male E3FAD mice.

### CS treatment reduces Aβ-pathology in male E3FAD mice

**Fig. 3:**
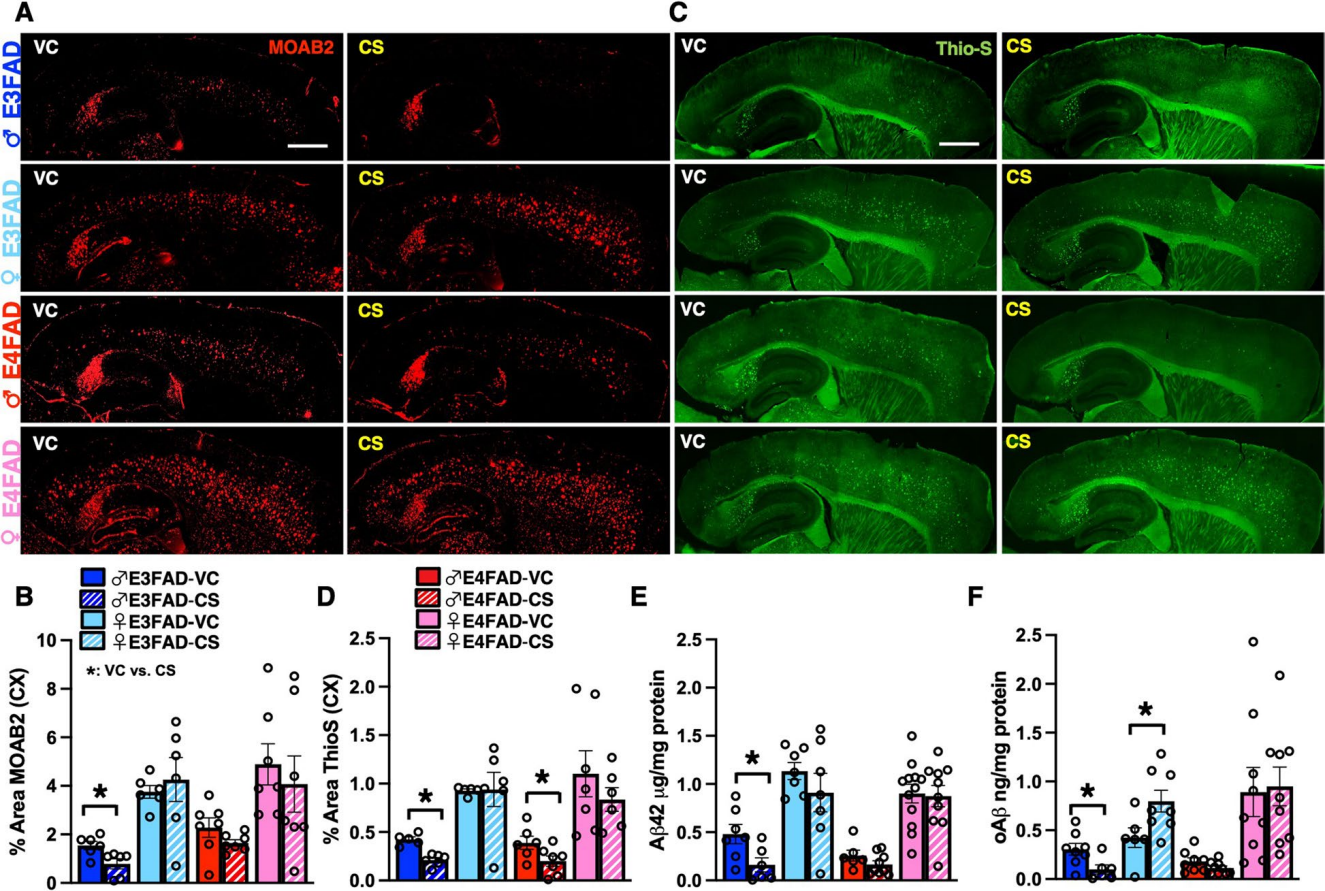
CS treatment reduces Aβ-pathology in male E3FAD mice. Effect of CS treatment on Aβ-relevant pathology. VC, vehicle control. **A** Representative images of Aβ immunostaining (MOAB-2) in sagittal sections. Scale bar: 100 μm. **B** Quantification of % area covered by Aβ in the cortex by IHC. CS treatment reduced Aβ deposition in male E3FAD mice (*p* = 0.0194). **C** Representative images of Thio-S staining for amyloid deposition in sagittal sections. Scale bar: 100 μm. **D** Quantification of % area Thio-S coverage in the cortex. CS treatment reduced amyloid deposition in male E3FAD mice (*p* = 0.0002) and male E4FAD mice (*p* = 0.0495). Aβ levels were measured in TBS (soluble) and formic acid (insoluble) cortical extracts by ELISA. **E** Insoluble Aβ42 levels were reduced with CS treatment in male E3FAD mice (*p* = 0.0295). **F** Soluble oAβ levels were reduced in male E3FAD mice (*p* = 0.0244) and increased in female E4FAD mice (*p* = 0.0337) All biochemical experiments were conducted in the cortex. Data are expressed as mean ± SEM (Biochem, *n* = 7–12; IHC, *n* = 6–7), analyzed by *t*-test, *: *p* < 0.05 CS vs. VC. All data is from the same cohort of mice; the *n*’s differ because of study design, technical issues, and outlier tests

Aβ pathology is considered an important contributing factor to AD progression, and CS has been proposed to regulate Aβ levels [24, 46, 47]. Therefore, we measured extracellular Aβ levels by immunohistochemical (IHC) analysis (MOAB-2), and fibrillar amyloid deposits by staining with Thio-S. CS treatment reduced Aβ and amyloid deposits in male E3FAD mice by ~ 60% (Fig. 3A–D). Consistent with this data, insoluble Aβ levels (formic acid) were reduced by ~ 70% with CS treatment in male E3FAD mice (Fig. 3E). In addition to extracellular deposits, soluble oligomeric Aβ (oAβ) is thought to be particularly detrimental in AD [48–51]. Importantly, we found that soluble oAβ levels were reduced with CS treatment by ~ 70% in male E3FAD mice (Fig. 3F). Interestingly, CS treatment reduces amyloid deposition but not any other Aβ measure in male E4FAD mice. In summary, CS treatment resulted in a reduction in Aβ pathology only in male E3FAD mice.

### CS treatment improves memory performance in MWM and increases synaptic protein levels of male E3FAD mice in the early paradigm

**Fig. 4:**
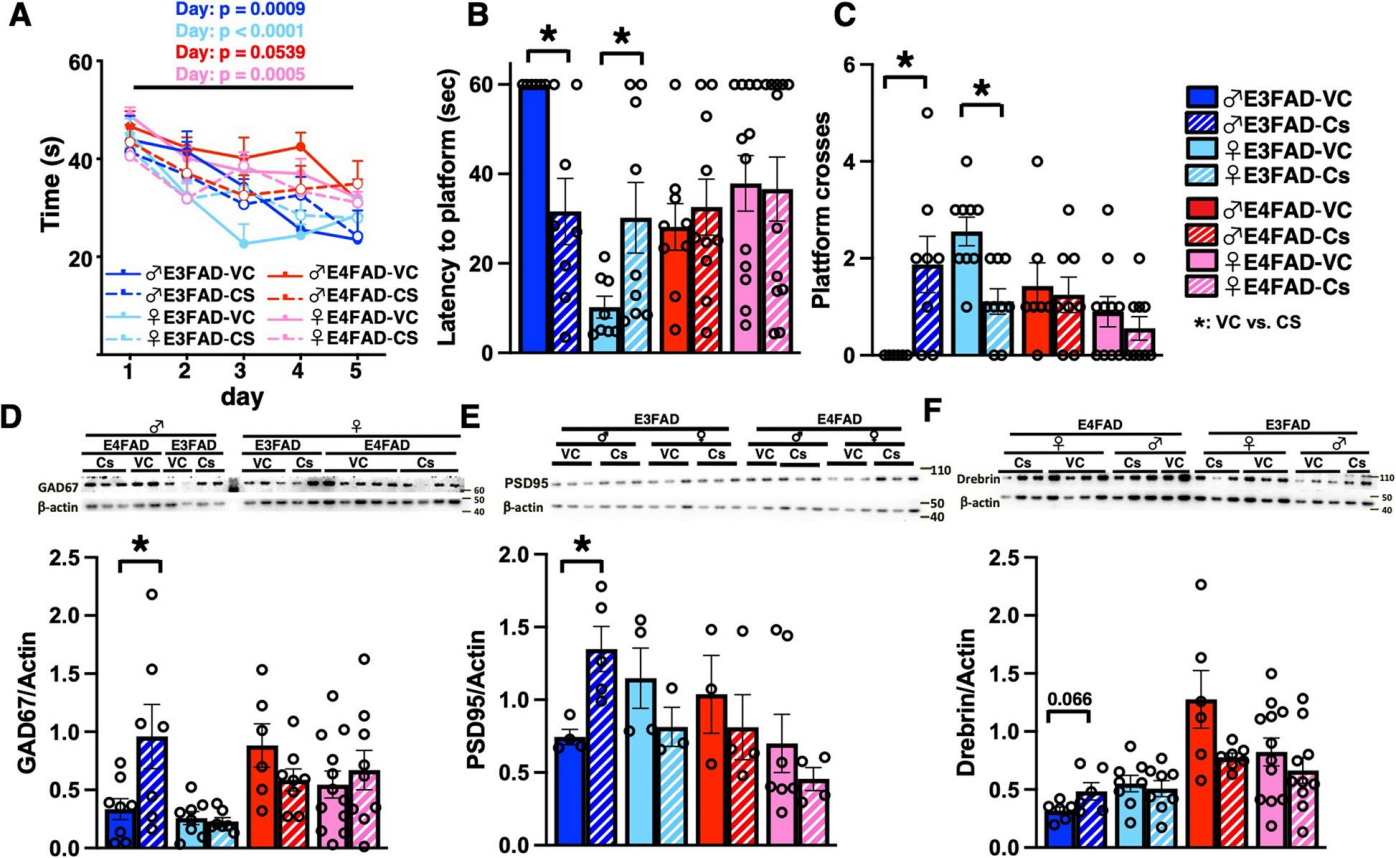
CS treatment improves memory-relevant behavior and levels of synaptic proteins in male E3FAD. Acquisition (**A**) and probe (**B** and **C**) trials in the Morris water maze test. In the acquisition phase, there was an effect of the day but not treatment in all groups. **B **and** C** Probe/memory trial performance. CS treatment in male E3FAD mice resulted in a lower latency to platform (*p* = 0.0064) (**B**) and a higher number of platform crosses (*p* = 0.0170) (**C**). CS treatment in female E3FADs resulted in higher latency to platform (*p* = 0.03666) (B) and lower number of platform crosses (*p* = 0.0020) (**C**). **D**–**F** Synaptic protein levels quantified by western blot. In male E3FAD mice, CS treatment resulted in higher levels of **D** GAD67 (*p* = 0.0408), **E** PSD95 (*p* = 0.0326), and, although non-significant, **F** drebrin levels (*p* = 0.066). Data are expressed as mean ± SEM for behavior (*n* = 8–10) and western blot (*n* = 5–12). MWM acquisition data were analyzed by 2-way ANOVA for treatment and day of test, followed by Tukey’s post hoc. Probe and western blot data were analyzed by by *t*-test, *: *p* < 0.05 CS vs. VC. Synaptic proteins were measured in the cortex. All data is from the same cohort of mice; the n’s differ because of study design, technical issues, and outlier tests

Since early treatment of CS lowered markers of Aβ-pathology, we next evaluated the impact on learning/memory using the MWM test. A limitation of the protocol employed in this manuscript is that we did not include a flag trial to check for blindness or differences in locomotor ability. However, we did not notice any overt signs of blindness (e.g., lack or responsive to guiding on the platform or to following hand movements) and in the probe trial there were no differences in swim speeds (Supplementary Fig. 3A). In the acquisition phase, overall, performance seemed greater with *APOE3* than *APOE4* but there was no effect of treatment (Fig. 4A). In the probe trial (memory), CS treated male E3FAD mice had lower latency to the platform (Fig. 4B) and an increase in the number of platform crosses (Fig. 4C). Our finding of apparent learning in the acquisition phase and lack of memory in the probe trial after CS treatment may be related to several factors. One is that in the acquisition phase of MWM, there are four trials each day, and the performance is averaged, whereas in the probe trial phase, there is only one trial. Therefore, mice may show some improvement in short-term learning/memory each day with repeated trials, but not in longer-term memory (24 h later), as we have observed in other treatment studies [42, 52–54]. The lack of memory is reflected in the track plots, where the treatment mice show more directed search strategies than non-treated mice towards the platform (Supplementary Fig. 3B). Alternatively, the differences in performance may be related to the specific protocol employed, and one of the future goals to compare different MWM protocols and how they impact learning and memory in EFAD mice. Alteration in levels of synaptic proteins often correspond to behavioral improvements [55]. We found that CS treatment increased levels of two important synaptic markers (GAD67 and PSD-95) in male E3FAD mice (Fig. 4D–E).

### Intraperitoneal treatment affects percentage survival in male EFADs and behavioral measures in male E3FAD mice

**Fig. 5:**
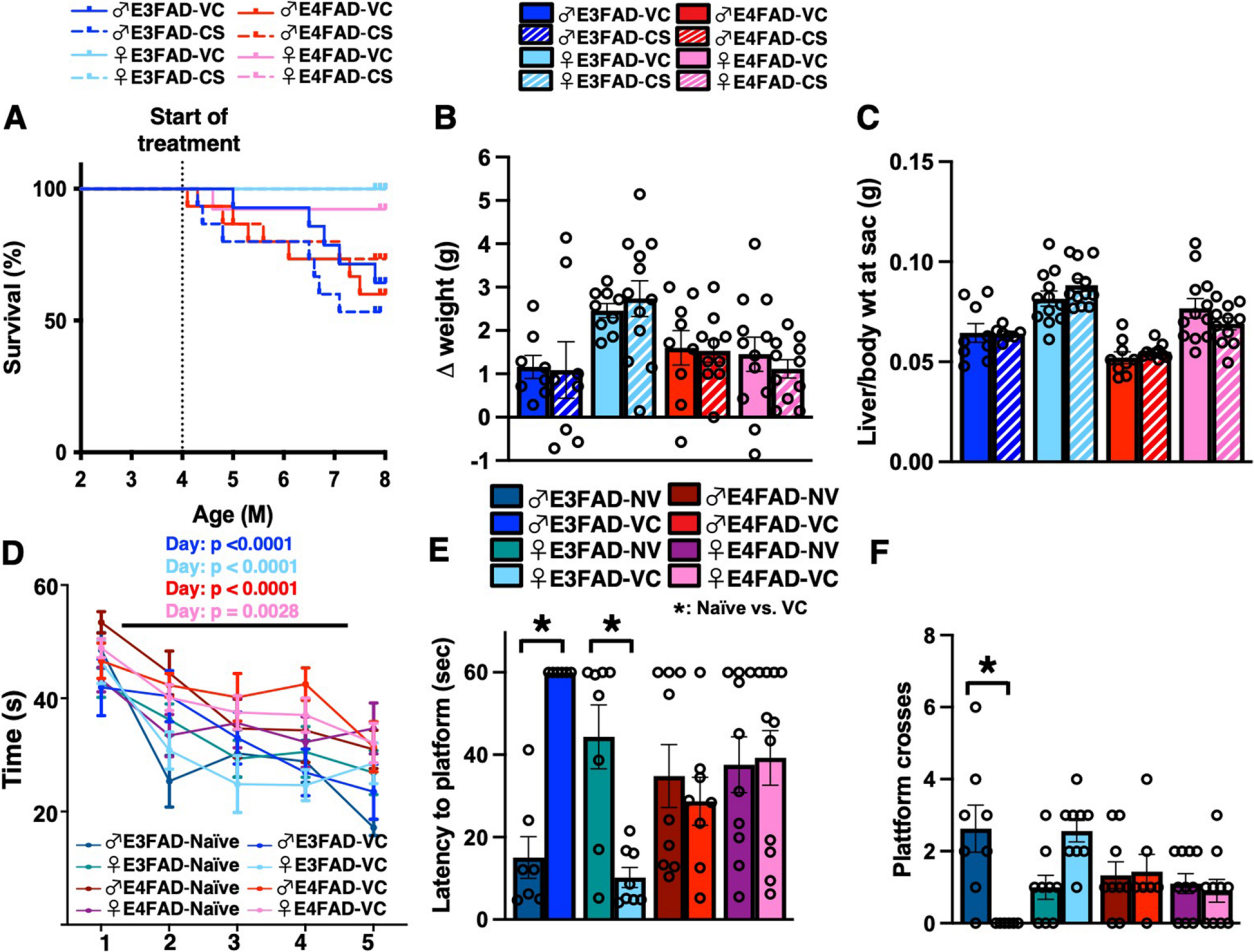
Intraperitoneal treatment affects % survival in male EFAD mice and behavior in male E3FAD mice. The % survival was determined after 4 months of chronic intraperitoneal treatment (i.p.) with vehicle control (VC) or CS in EFAD mice **A** There were no differences in % of survival due to treatment (*p* = 0.8747), or *APOE* genotype (0.7860). However, there was a reduction in % survival with sex (*p* < 0.0001), as less males survived compared to females. CS treatment did not affect **B** body or **C** Liver weights. To evaluate the effect of chronic i.p. treatment on behavioral measurements, we compared VC-treated mice versus a naïve cohort of 8-month-old EFAD mice in the Morris water maze test. **D** In the Acquisition phase there was an effect of day but not treatment in all groups. **E** and **F** Probe/memory trial performance. In E3FAD mice, compared to the naïve cohort, chronic i.p. injections resulted in higher latency to platform (*p* < 0.0001) (**E**) and lower number of platform crosses (*p* = 0.0026) (**F**). Data are expressed as mean ± SEM (*n* = 9–12); *: *p* < 0.05 CS vs. VC. Percentage survival was analyzed by chi-square (Mantel-Cox) test. MWM acquisition data were analyzed by 2-way ANOVA, followed by Tukey’s post hoc. The rest of the data was analyzed by by *t*-test, *: *p* < 0.05 CS vs. VC. All data is from the same cohort of mice; the *n*’s differ because of study design, technical issues, and outlier tests

We were somewhat surprised that visually, vehicle-treated male E3FAD mice appeared impaired in the probe trial of the MWM test compared to all other groups including E4FAD mice. We therefore considered that daily i.p. treatment for 4 months may have affected MWM performance in male E3FAD mice. In fact, during the study, although we didn’t find an effect of CS treatment on the percentage survival rate (Fig. 5A), we did find higher death rates in males vs. females (no effect on body or liver weights; Fig. 5B and C). Therefore, we repeated our MWM protocol with a naïve cohort and compared the data to mice that had received chronic VC i.p. treatment. We found that compared to the naïve cohort, chronic i.p. injections resulted in higher latency to platform and a lower number of platform crosses in male E3FAD mice (Fig. 5D–F). These data imply that CS was able to perhaps mitigate the behavioral dysfunction caused by chronic daily i.p. injections in male E3FAD mice.

### CS treatment of EFAD mice at ages of high pathology does not impact ABCA1/apoE levels, AD-like pathology, or behavior

**Fig. 6:**
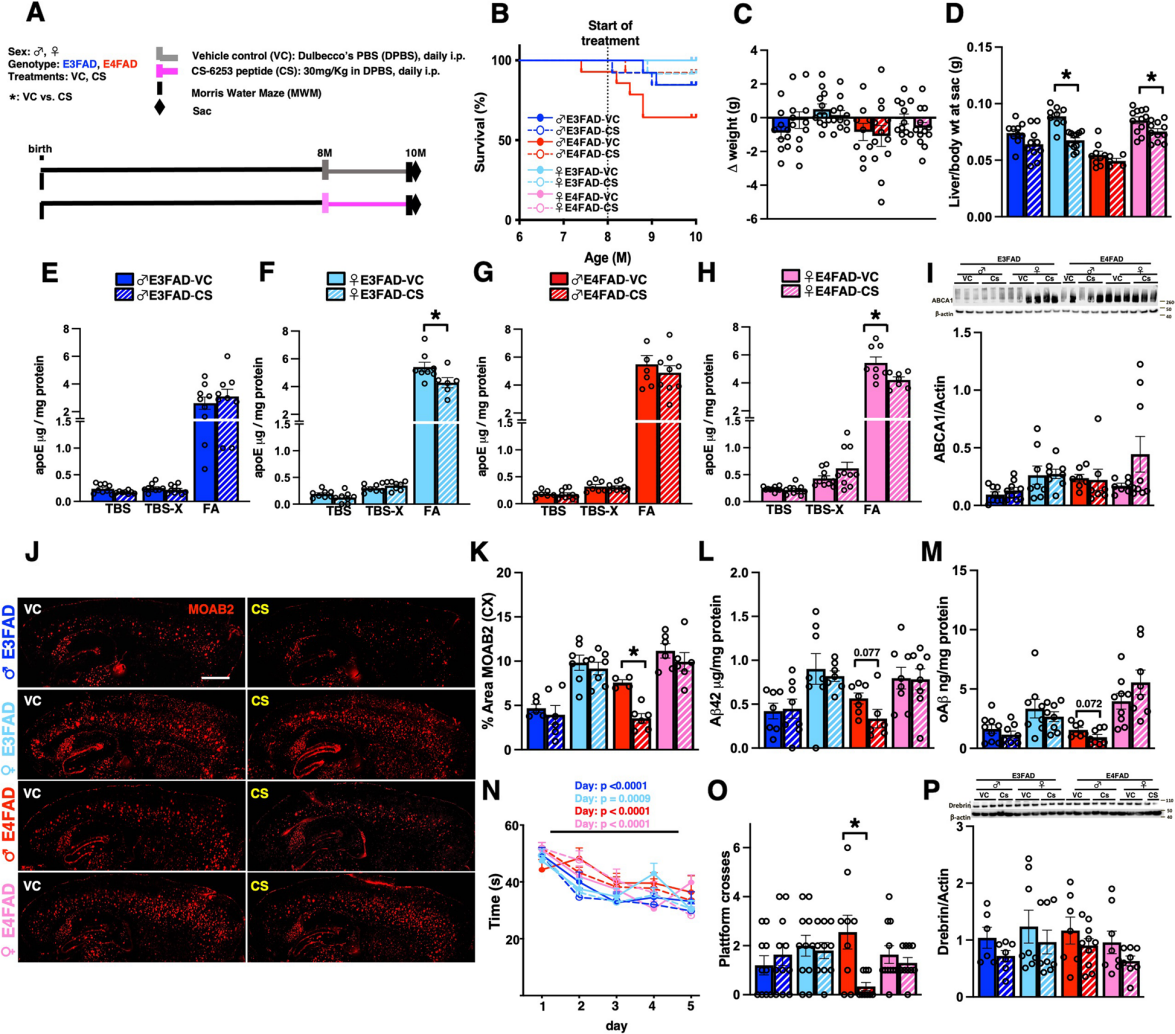
CS treatment of EFAD mice at ages of high pathology does not impact AD-like phenotype to a great extent. **A** Study design. EFAD mice were treated with CS or VC by daily intraperitoneal injections (i.p.) from 8 to 10 months of age. **B** There were no differences in % of survival due to treatment (*p* = 0.8422), or *APOE* genotype (*P* = 0.7353), however males survived less compared to females (*p* < 0.0069). **C** CS treatment did not affect body weight. **D** Liver weights were reduced by CS treatment in female mice (E3FADs, *p* < 0.0001; E4FADs, *p* = 0.0227). **E**–**H** CS treatment did not modulate soluble or detergent-soluble apoE. **F** and **H** CS treatment decreased insoluble apoE in female EFAD mice (E3FAD, *p* < 0.0472; E4FAD, *p* = 0.0291).** I** There were no effects of CS treatment on ABCA1 levels. **J** Representative images of Aβ immunostaining (MOAB-2) in sagittal sections. Scale bar: 100 μm. **K** CS reduced % area covered by Aβ in male E4FAD (*p* = 0.0005). **L** Soluble oAβ levels were not affected by CS treatment except for a non-significant trend in male E4FAD mice (*p* = 0.077). **M** Insoluble Aβ levels were not affected by CS treatment except for a non-significant trend in male E4FAD mice (*p* = 0.072). Acquisition (**N**) and probe (**O**) trials in the Morris water maze (MWM) test. In the acquisition phase, there was an effect of day but not treatment in all groups. **O** Probe/memory trial performance. There was a reduction in platform crosses with CS treatment in male E4FAD (*p* = 0.0064). **P** CS treatment did not affect drebrin levels. Data are expressed as mean ± SEM for behavior, body weights, and biochemical measures (*n* = 9–12); and IHC and western blot (*n* = 6–8). MWM acquisition data were analyzed by repeated measures using 4-way ANOVA, followed by Tukey’s post hoc. % survival was analyzed by chi-square (Mantel-Cox) test. The rest of the data was analyzed by *t*-test. All data is from the same cohort of mice; the n’s differ because of study design, technical issues, and outlier tests

Male E3FAD mice were the only group that consistently responded to CS treatment and when treatment was initiated (4 months of age) had the lowest amount of Aβ pathology (Supplementary Fig. 2). One explanation for the E3FAD male-specific beneficial effects is that levels of Aβ pathology could determine the efficacy of CS treatment. Therefore, we treated EFAD mice from 8 to 10 months of age (Fig. 6A) when pathology was fully established for all the groups (Fig. 3-VC). Treatment with CS did not affect % survival or change of body weights (Fig. 6B and C); however, it reduced liver weights in the female mice when normalized by body weight (Fig. 6D). We next measured readouts of indirect target engagement and found that CS treatment had no effect on levels of ABCA1, soluble apoE, detergent-soluble apoE or ABCA1 levels in any of the groups (Fig. 6E–I). Interestingly, CS treatment reduced insoluble apoE levels in female EFAD mice (Fig. 6F and H). Regarding Aβ pathology and behavioral measures, there was not a clear effect by CS treatment. In general, CS treatment did not change Aβ deposition, soluble and insoluble Aβ levels (Fig. 6J–M), or behavioral/synaptic readouts (Fig. 6N–P). Intriguingly, there was a reduction of Aβ deposition that was not accompanied by a reduction on Aβ levels or an improvement in behavior in male E4FAD mice (Fig. 6J–P). Therefore, CS treatment of EFAD mice at ages of high Aβ levels did not reduce AD-relevant pathology in male E3FAD mice.

## Discussion

ABCA1 has been suggested as a therapeutic approach to increase apoE levels and/or lipidation in the brain and therefore lower Aβ levels and other pathology to improve neuron function and cognition in AD patients. Evidence for this approach includes that ABCA1 loss-of-function mutations increase AD risk [56] and the reduced ability of lipoproteins isolated from the cerebrospinal fluid (CSF) of AD patients to accept cholesterol from ABCA1 compared to lipoproteins from age-matched controls [57]. In addition, ABCA1 knockout increases and ABCA1 overexpression reduces Aβ pathology [33, 34]. These human and genetic studies led to a focus on identifying compounds that activate ABCA1. To date, most efforts have centered on transcriptional upregulation of *APOE* and ABCA1 via nuclear receptor agonists (NR) as a therapy for AD [58, 59]. NRs such as LXR and RXR alter the expression of multiple genes involved in inflammation and importantly, lipid metabolism including *APOE* and ABCA1 [60]. In general, agonists of the nuclear receptors RXR (e.g., bexarotene), LXR, and PPARγ have been shown to increase ABCA1 levels, increase apoE levels/lipidation, and reduce Aβ pathology and cognition in familial AD (FAD) mice [25, 61–67]. However, there are concerns of the side effects of NR receptor agonists including induction of hepatic lipogenesis leading to hepatomegaly [25, 68] that could explain in part the lack of reproducibility [69–73]. In fact, in E4FAD mice we found bexarotene treatment lowered Aβ levels in the hippocampus but not the cortex possibly by induced hepatic encephalopathy [25]. To circumvent these issues, researchers are developing novel NR agonists that specifically increase brain apoE levels/lipidation without detrimental peripheral side effects [66, 74]. An alternative approach is to directly target ABCA1 activity. CS-6253 was developed to stabilize ABCA1, prevent its degradation, and facilitate translocation of ABCA1 to the plasma membrane in turn increasing cholesterol efflux to acceptor lipoproteins [36, 75]. We found that in young male E3FAD mice, CS increased brain apoE levels (soluble and lipid associated) without an induction of hepatomegaly resulting in lower Aβ pathology and improved cognition as well as markers of neuron function and memory. Although there are caveats (see below), our data in EFAD mice support the overall concept that targeting ABCA1 is a potential therapy for *APOE3* carriers at early stages of Aβ pathology.

Increasing apoE levels and/or lipidation via ABCA1 is proposed as particularly beneficial for *APOE4* carriers. Indeed, *APOE4* is associated with low levels of apoE in the brain, CSF, and plasma in healthy controls and AD patients compared to *APOE3* [76]. In addition, apoE4-lipoproteins are less lipidated and preferentially degraded compared to apoE3-lipoproteins in vitro and in vivo [17, 32, 77]. Human apoE4-lipoproteins also have a reduced ability to accept cholesterol from ABCA1 compared to apoE3-lipoproteins when isolated from human CSF [47]. Lower apoE4 lipidation is thought to result in several functional consequences, including less efficient clearance of Aβ compared to apoE3 [78]. Thus, therapeutic efforts have focused on targeting apoE4 lipoproteins to resemble apoE3-lipoproteins. In fact, NR agonists have been shown to lower Aβ levels in mice that express apoE4 including E4FAD mice [25, 62]. Therefore, targeting ABCA1 activation is also proposed to be beneficial for *APOE4* carriers in AD. In support, CS-6253 has been demonstrated to increase apoE4 lipidation, reduce intracellular Aβ42 levels, and improve cognitive performance in α-synuclein-KO/*APOE4-targeted replacement* mice [24]. However, in this current study, CS-6253 treatment did not modify apoE4-lipoproteins, Aβ levels or behavior in the E4FAD mice. One potential explanation is that the combination of *APOE4* and Aβ elevations in the E4FAD model disrupts lipid metabolism to such an extent that ABCA1 stabilization by CS-6253 is no longer sufficient to increase apoE4 lipidation. Previous studies have found that *APOE4* is independently associated with lipid dysregulation including impaired ABCA1 translocation to the plasma membrane [47], reduced fatty acid transport from neurons to astrocytes [79], and lipid droplet accumulation in astrocytes [80] and microglia [81]. Aβ has also been found to disrupt lipid metabolism, including limiting cholesterol availability. For example, Aβ induces lipid droplet accumulation in glia [82, 83], which would reduce the availability of free cholesterol for efflux. Aβ has also been shown to upregulate cholesterol biosynthesis via increasing 3-hydroxy-3-methyl-glutaryl-coenzyme A reductase (HMGCoA) and ABCA1 in glia [84, 85], without improving cholesterol efflux [86] suggesting a direct interaction between Aβ and ABCA1 [84]. In addition, soluble Aβ oligomers can interact with lipid membranes, compromising its integrity and function [40, 87–90], which may interfere with ABCA1 activity. Thus, the combination of high Aβ levels and *APOE4* may have impeded lipid metabolism and therefore the ability of CS to be beneficial for *APOE4* mice. Indeed, in this study, ABCA1 and Aβ levels appeared higher in E4FAD mice, potentially as a compensatory mechanism and therefore it may not be possible to further increase ABCA1 activity. In addition, previous studies demonstrating the beneficial effects of targeting ABCA1 were in models or ages of low Aβ pathology and normal Aβ production [24, 25, 91]. Therefore, utilizing Aβ directed co-therapies when targeting cholesterol metabolism and ABCA1 in *APOE4* carriers may be beneficial.

## Limitations

We are limited in the extent that we can draw conclusions on whether CS is beneficial for a specific *APOE* genotype, sex, or stage of pathology that could be addressed in future studies. Although we propose that the stage of pathology determines CS activity due to lipid dysregulation, it is important to repeat our study with CS treatment initiated at younger ages. Further, the concentration/dose and treatment regime may need optimizing using a combination of pharmacokinetic (PK) and in vitro studies. In terms of PK, data are limited on the extent that CS is brain penetrant partially due to technical difficulties [46]. *APOE* genotype also needs to be incorporated into future CS PK studies, since *APOE4* disrupts brain endothelial cell function including permeability. Related, it is unclear what the target concentration of CS is in the brain for its beneficial effects. CS dose-dependently increases apoE and cholesterol efflux in vitro [36] (Fig. 1C, D). We also found that at lower CS concentrations, close to the EC50 value for ABCA1-mediated cholesterol efflux [37], apoE lipidation is efficient while at 10- and 100-fold higher CS concentrations there is no impact on apoE lipidation (Supplementary Fig. 4) indicating the importance of identifying the correct dosing regimen. In addition to the dose, the route of administration may act as a cofounder for the evaluation of the beneficial effects of CS on behavior. In this study, daily i.p. injections resulted in behavioral impairments in the male E3FAD mice that was mitigated by the CS treatment. Therefore, alternative treatment methods would be complementary to fully understand if CS can produce a long-lasting beneficial effect on learning and memory. Interestingly CS administration in cynomolgus monkeys increased plasma apoE, small HDL and Aβ42/40-ratio, effects that were sustained through 30 days of treatment [46]. CS is now in phase 1 SAD-MAD studies in elderly men and women with and without *APOE4* genotype (NCT05965414).

Future studies could also incorporate expanded readouts for target engagement and activity. For a full evaluation of target engagement, in addition to apoE levels and lipidation, additional readouts could be incorporated for ABCA1 activity and aggregation, as well as global and cellular lipid metabolism including lipidomics, lipid droplets, and plasma membrane fluidity. Further, the effects of modulating ABCA1 by CS-6253 on myelination were recently reported [92] and may shed light on other AD-relevant pathology including neuroinflammation, neuronal activity, and complementary behavioral assays.

## Conclusion

CS treatment reduced Aβ pathology and improved memory in young male mice that express *APOE3* but not in female *APOE3* mice or mice that express *APOE4*. Therefore, levels of Aβ pathology may impact the ability of targeting only ABCA1 to be an effective Alzheimer’s disease therapeutic.

### Supplementary Information

**Additional file 1:** **Supplementary Table 1.** Antibodies and kits information.

**Additional file 2: Supplementary Figure 1.** In vitro supplementary data. A. Raw MTT values of *APOE4*-glia in response to 6h or 24h treatment with increasing concentrations of CS (0.0-500 μg/ml). C. Raw values of apoE ELISA with either *APOE-KO* or *APOE3-glia* (media) in response to 24hr CS treatment (0.0-500.0 μg/ml). Data are expressed as means ± SEM (*n*=4) and analyzed by t-test.

**Additional file 3: Supplementary Figure 2.** EFAD mice exhibit sex- and *APOE*-dependent AD-relevant phenotypes at 4 months of age. AD-relevant phenotypic readouts in a naïve cohort of 4-month-old E3FAD (*APOE3*) and E4FAD (*APOE4*) mice. A. ABCA1 levels quantified by western blot (representative image). There was no effect of *APOE* genotype (*p*=0.3393) but a trend of sex (*p*=0.0501) on ABCA1 levels. B. Quantification of apoE native particles: high molecular weight particles (large particles, LP, left); intermediate molecular weight particles (intermediate particles, IP, middle) and low molecular weight particles (small particles, SP, right) by native blots. The relative amount of apoE in HMW particles was greater with *APOE3* compared to *APOE4* [F_(1,17)_: 4.464, *p*=0.0497]. ApoE levels were measured in the cortex after sequential homogenization with TBS (soluble), then 1% TritonX100 (detergent soluble), followed by formic acid (insoluble) by ELISA. C. Total apoE levels. There was an interaction between sex and *APOE* genotype [Sex x genotype interaction: F_(1,19)_: 4.445, *p*=0.0485] that was due to higher levels in female *APOE4* mice compared to both male groups. D. There were higher soluble apoE levels in female mice than males [F_(1,19)_: 15.52, *p*=0.009]. E. There were higher detergent-soluble apoE levels with *APOE3* compared to*APOE4* [F_(1,18)_: 4.811, *p*=0.0417]. F. Insoluble apoE levels were higher with *APOE4* and female sex [Sex: F_(1,18)_: 8.118, *p*=0.0106; Genotype: F_(1,18)_: 8.186, *p*=0.0104]. G. Representative images of Thio-S staining for amyloid deposition in sagittal sections. Scale Bar: 100 μm. H. Quantification of % area Thio-S coverage in the cortex. There was higher amyloid deposition with *APOE4* and female sex [Sex: F_(1,21)_: 12.46, *p*=0.0020; Genotype: F_(1,21)_: 11.83,*p*=0.0225]. I. Representative images of Aβ immunostaining (MOAB-2) in sagittal sections. Scale Bar: 100 μm. J. Quantification of % area covered by Aβ in the cortex by IHC. Aβ deposition was higher in female mice [Sex: F_(1,21)_: 6.152, *p*=0.0217]. Aβ42 levels were measured in TBS (soluble) and formic acid (insoluble) cortical extracts by ELISA. K. Soluble Aβ42 levels were higher with *APOE4* compared to *APOE3* [F_(1,26)_: 7.050, *p*=0.0134]. L. Insoluble Aβ42 levels were higher in *APOE4* females than all other groups [Sex x genotype interaction: F_(1,17)_: 7.574,*p*=0.0136]. Acquisition (M) and probe trial (N&O) in a modified Morris water maze test. M. There was a day and genotype interaction [Sex x genotype interaction: F_(4,165)_= 2.754, *p*=0.0298] because E4FAD latencies are higher at day 5 (*p*=0.0328). N&O. Probe/memory trial latencies. Perfomance in the probe trial was better with *APOE3* as indicated by a lower latency to plattform [F_(1,34)_: 7.372, *p*=0.0103] (N) and higher number of plattform crosses [F_(1,33)_: 6.915, *p*=0.0206] (O). P. PSD95 levels quantified by western blot. There is an interaction of sex and genotype [F_(1,8)_: 5.889, *p*=0.0414] because females E3FAD are higher than the rest of the groups. All biochemical readouts were performed in the cortex. Data are expressed as mean ± SEM (*n*=3-5 for western blot and native gels; *n*=6-8 for biochemistry and IHC and *n*=6-12 for behavior), analyzed by 2-way ANOVA for sex, *APOE* genotype and their interaction. MWM acquisition data were analyzed by repeated measures using 3-way ANOVA, followed by Tukey’s post hoc. Probe and western blot data was analyzed by by t-test, *: *p*<0.05 vs. vehicle (VC). All data is from the same cohort of mice; the n’s differ due to data and tissue not available, technical issues, and outlier tests.

**Additional file 4: Supplementary Figure 3.** Behavior supplementary data (male E3FAD mice only). A. Total swim speed in probe trial. B. Probe trial track plots.

**Additional file 5: Supplementary Figure 4. **CS facilitates apoE4 lipidation at 0.5 μg/mL. *APOE4-*mixed glial were treated with CS-6253 (CS) (0.5-50 μg/ml) or vehicle control (VC) for 24h and secreted apoE particles were analyzed by native gels. Large (LP) and intermediate particles (IP) are increased with 0.5 μg/mL of CS treatment.

## Data Availability

The datasets analyzed during the current study are available from the corresponding author on reasonable request.

## Abbreviations

AD: Alzheimer’s disease
Aβ: Amyloid-beta peptide
ABCA1: ATP-binding cassette transporter A1
*APOE*: Apolipoprotein E gene
apoE: Apolipoprotein E protein
CNS: Central nervous system
CSF: Cerebrospinal fluid
CS: CS-6253 peptide
ELISA: Enzyme-linked immunosorbent assay
IHC: Immunohistochemistry
FAD: Familial AD
GAD67: Glutamate decarboxylase isoform 67
MTT: 3-(4,5-Dimethylthiazol-2-yl)-2,5-diphenyltetrazolium bromide
MWM: Morris water maze
oAβ: Oligomeric Aβ
PSD95: Postsynaptic density protein 95
PBS: Phosphate-buffered saline
TBS: Tris-buffered saline
TBSX: Tris-buffered saline with Triton-X 1%
FA: Formic acid
Thio-S: Thioflavin-S
VC: Vehicle control
NR: Nuclear receptor
