# Supplementary material for: A novel apoE-mimetic increases brain apoE levels, reduces Aβ pathology and improves memory when treated before onset of pathology in male mice that express APOE3
Source: Alzheimers Res Ther. 2023 Dec 15;15:216. doi: 10.1186/s13195-023-01353-z (PMC10722727; doi:10.1186/s13195-023-01353-z)
Supplement: Supplementary file 1 — Additional file 1: Supplementary Table 1. Antibodies and kits information. [file 13195_2023_1353_MOESM1_ESM.docx]

| **Reagent** | **Catalog #** | **Vendor** | **Dilution** | **Duration** | **Temp** | **Application** |
| --- | --- | --- | --- | --- | --- | --- |
| 𝝰-Aβ: MOAB-2 |  | In-house antibody (stock at at 1mg/mL) | 1:500 | O/N | 4℃ | IHC |
| anti-Mouse IgG | A31571 | ThermoFisher | 1:250 | 2 hr | RT |  |
| anti-Rabbit IgG | A11034 | ThermoFisher | 1:250 | 2 hr | RT |  |
| apoE in-house: 𝝰-apoE (coating) | AB947 | MilliporeSigma | 1:2000 | O/N | RT | BC |
| apoE in-house: 𝝰-apoE (detection) | K74180B | Meridian Life Science | 1:5000 | O/N | 4℃ | BC |
| apoE in-house: apoE (STD) | ApoE3-3562H | Creative BioMart Inc. | 200 ng/mL | - | - | BC |
| Human Aβ42 kit | KHB3442 | ThermoFisher | - | - | - | BC |
| Oligomeric Aβ kit | BEK-2215-1P | Biosensis | - | - | - | BC |

**Supplementary Table 1.** Antibodies and kits information**.**
