# Supplementary figures and images for: A novel apoE-mimetic increases brain apoE levels, reduces Aβ pathology and improves memory when treated before onset of pathology in male mice that express APOE3
Source: Alzheimers Res Ther. 2023 Dec 15;15:216. doi: 10.1186/s13195-023-01353-z (PMC10722727; doi:10.1186/s13195-023-01353-z)

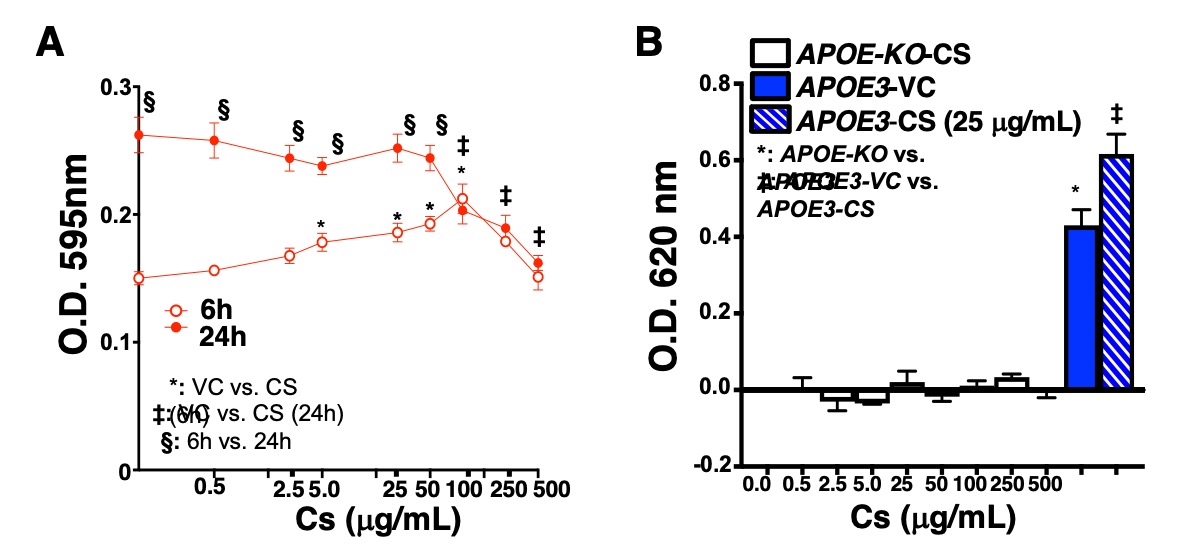

Supplement: Supplementary file 2 — Additional file 2: Supplementary Figure 1. In vitro supplementary data. A. Raw MTT values of APOE4-glia in response to 6h or 24h treatment with increasing concentrations of CS (0.0-500 μg/ml). C. Raw values of apoE ELISA with either APOE-KO or APOE3-glia (media) in response to 24hr CS treatment (0.0-500.0 μg/ml). Data are expressed as means ± SEM (n=4) and analyzed by t-test. [file 13195_2023_1353_MOESM2_ESM.jpg]

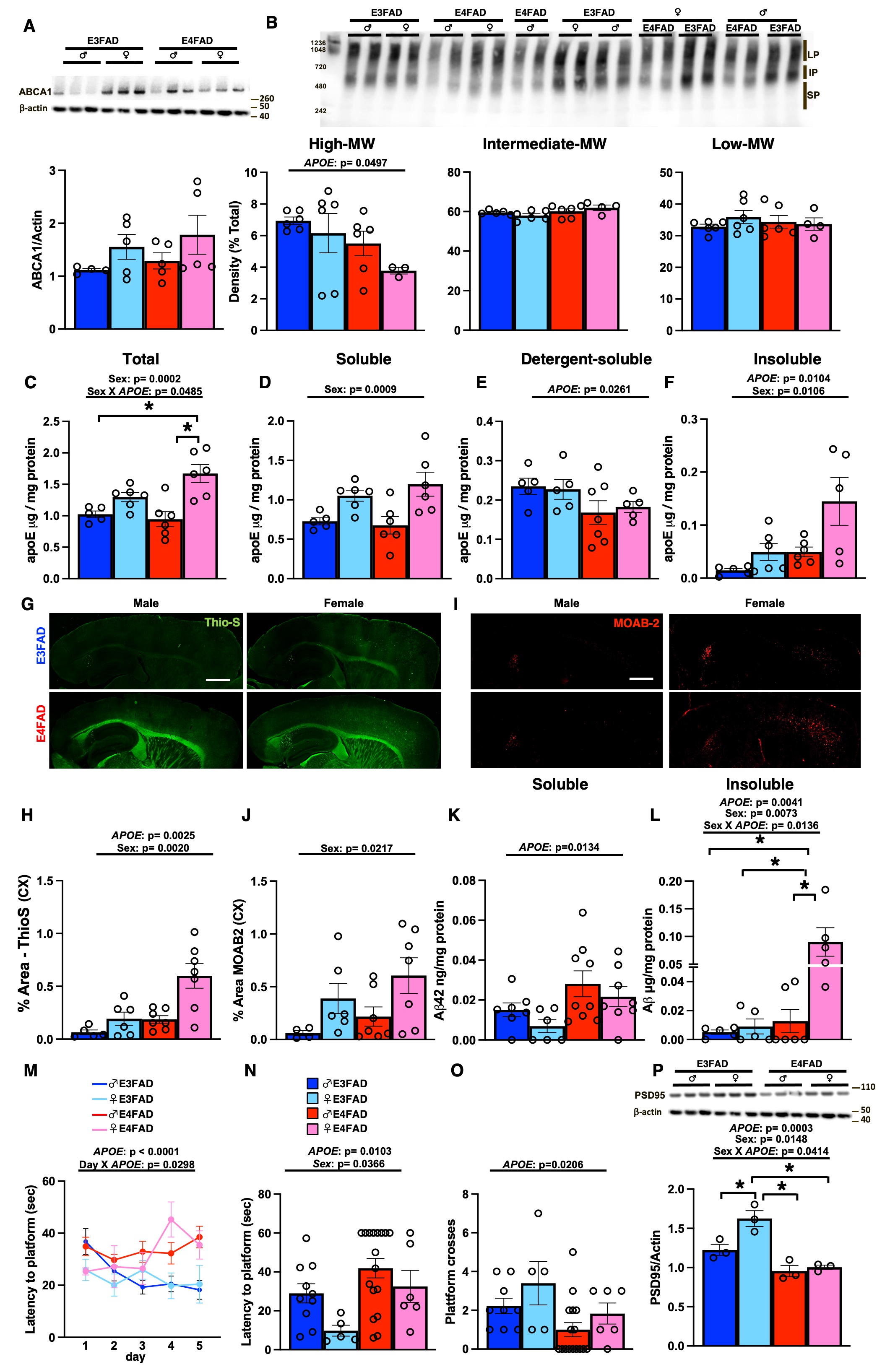

Supplement: Supplementary file 3 — Additional file 3: Supplementary Figure 2. EFAD mice exhibit sex- and APOE-dependent AD-relevant phenotypes at 4 months of age. AD-relevant phenotypic readouts in a naïve cohort of 4-month-old E3FAD (APOE3) and E4FAD (APOE4) mice. A. ABCA1 levels quantified by western blot (representative image). There was no effect of APOE genotype (p=0.3393) but a trend of sex (p=0.0501) on ABCA1 levels. B. Quantification of apoE native particles: high molecular weight particles (large particles, LP, left); intermediate molecular weight particles (intermediate particles, IP, middle) and low molecular weight particles (small particles, SP, right) by native blots. The relative amount of apoE in HMW particles was greater with APOE3 compared to APOE4 [F(1,17): 4.464, p=0.0497]. ApoE levels were measured in the cortex after sequential homogenization with TBS (soluble), then 1% TritonX100 (detergent soluble), followed by formic acid (insoluble) by ELISA. C. Total apoE levels. There was an interaction between sex and APOE genotype [Sex x genotype interaction: F(1,19): 4.445, p=0.0485] that was due to higher levels in female APOE4 mice compared to both male groups. D. There were higher soluble apoE levels in female mice than males [F(1,19): 15.52, p=0.009]. E. There were higher detergent-soluble apoE levels with APOE3 compared toAPOE4 [F(1,18): 4.811, p=0.0417]. F. Insoluble apoE levels were higher with APOE4 and female sex [Sex: F(1,18): 8.118, p=0.0106; Genotype: F(1,18): 8.186, p=0.0104]. G. Representative images of Thio-S staining for amyloid deposition in sagittal sections. Scale Bar: 100 μm. H. Quantification of % area Thio-S coverage in the cortex. There was higher amyloid deposition with APOE4 and female sex [Sex: F(1,21): 12.46, p=0.0020; Genotype: F(1,21): 11.83,p=0.0225]. I. Representative images of Aβ immunostaining (MOAB-2) in sagittal sections. Scale Bar: 100 μm. J. Quantification of % area covered by Aβ in the cortex by IHC. Aβ deposition was higher in female mice [Sex: F(1 [file 13195_2023_1353_MOESM3_ESM.jpg]

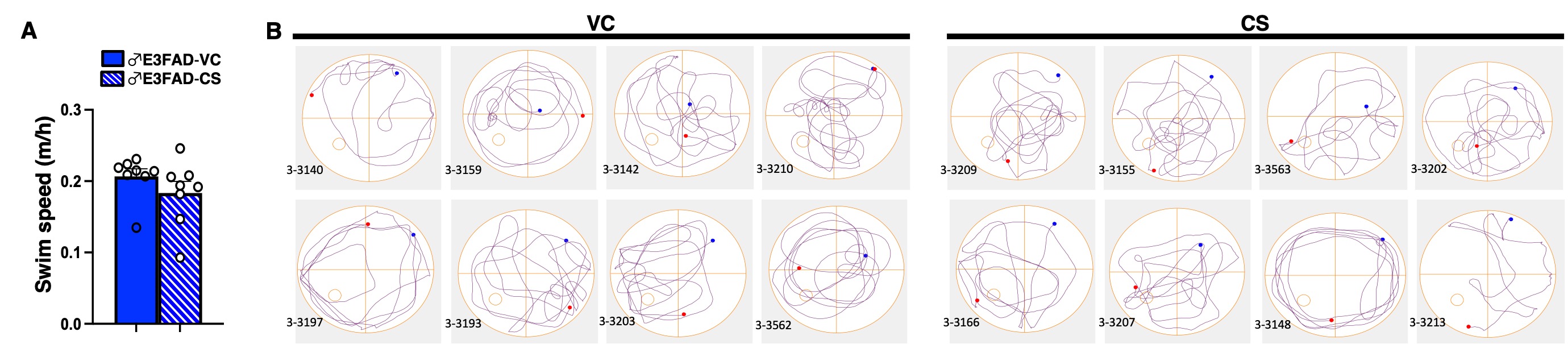

Supplement: Supplementary file 4 — Additional file 4: Supplementary Figure 3. Behavior supplementary data (male E3FAD mice only). A. Total swim speed in probe trial. B. Probe trial track plots. [file 13195_2023_1353_MOESM4_ESM.jpg]

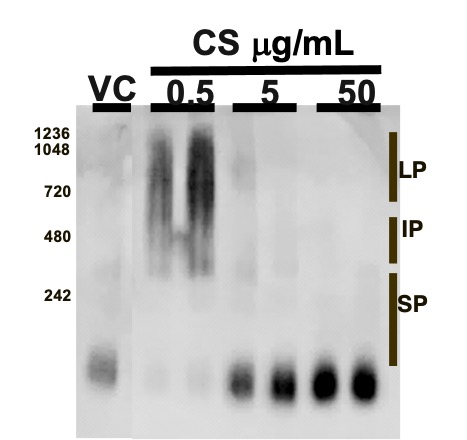

Supplement: Supplementary file 5 — Additional file 5: Supplementary Figure 4. CS facilitates apoE4 lipidation at 0.5 μg/mL. APOE4-mixed glial were treated with CS-6253 (CS) (0.5-50 μg/ml) or vehicle control (VC) for 24h and secreted apoE particles were analyzed by native gels. Large (LP) and intermediate particles (IP) are increased with 0.5 μg/mL of CS treatment. [file 13195_2023_1353_MOESM5_ESM.jpg]
